# Stepwise protein targeting into plastoglobules are facilitated by three hydrophobic regions of rice phytoene synthase 2

**DOI:** 10.3389/fpls.2023.1181311

**Published:** 2023-05-31

**Authors:** Ji Su Yu, Min Kyoung You, Yeo Jin Lee, Sun-Hwa Ha

**Affiliations:** Department of Genetics and Biotechnology, Graduate School of Green-Bio Science, College of Life Sciences, Kyung Hee University, Yongin, Republic of Korea

**Keywords:** carotenoid, cleavage site, hydrophobic helix, transmembrane domain, transit peptide

## Abstract

Plastoglobules (PGs) are plastidial lipid droplets enclosed by a polar monolayer born from the thylakoid membrane when plants require active lipid metabolism, including carotenogenesis, under the environmental stress and during plastid transition. Despite the fact that many proteins are reported to target PGs, their translocation mechanism has remained largely unexplored. To elucidate this process, we studied the influence of three hydrophobic regions (HR)—HR1 (1–45^th^ aa), HR2 (46–80^th^ aa), and HR3 (229–247^th^ aa)—of rice phytoene synthase 2 (OsPSY2, 398 aa), which has previously shown to target PGs. As results, HR1 includes the crucial sequence (31–45^th^ aa) for chloroplast import and the stromal cleavage occurs at a specific alanine site (64^th^ aa) within HR2, verifying that a N-terminal 64-aa-region works as the transit peptide (Tp). HR2 has a weak PG-targeting signal by showing synchronous and asynchronous localization patterns in both PGs and stroma of chloroplasts. HR3 exhibited a strong PG-targeting role with the required positional specificity to prevent potential issues such as non-accumulation, aggregation, and folding errors in proteins. Herein, we characterized a Tp and two transmembrane domains in three HRs of OsPSY2 and propose a spontaneous pathway for its PG-translocation with a shape embedded in the PG-monolayer. Given this subplastidial localization, we suggest six sophisticated tactics for plant biotechnology applications, including metabolic engineering and molecular farming.

## Introduction

Chloroplasts are organelles where photosynthesis occurs and that are vital for plant survival. They also participate in metabolic processes for carbohydrates, amino acids, lipids, vitamins, and isoprenoids (Menke, 1962; Chen et al., 2018). Chloroplasts consist of subcellular aqueous regions, such as the stroma and thylakoid lumen, and various membranous structures, including the outer and inner envelope, internal thylakoid membrane, and plastoglobules (PGs). PGs are lipid monolayer droplets associated with the thylakoid membranes (Bailey and Whyborn, 1963; Greenwood et al., 1963; Rottet et al., 2015). The proteins found in various chloroplast structures, excluding the outer envelope, possess a transit peptide (Tp) that is susceptible to cleavage in the precursor N-terminal region. The Tp facilitates protein localization from the cytoplasm to the stroma *via* consecutive traversal of two envelopes through one translocon at the outer envelope membrane of chloroplasts and another translocon at the inner envelope membrane of chloroplast complexes and then is cleaved by a stromal processing peptidase. The cleaved mature proteins have different fates: some remain in the stroma and some are embedded in the inner envelope or engage in secondary transportation-mediated translocation toward the thylakoid or thylakoid-derived structures such as PGs in the help of additional signals (Jarvis and Robinson, 2004; Jarvis, 2008; Paila et al., 2015; Xu et al., 2021).

To precisely sort proteins to thylakoidal structures, several independent routes have been identified, including the chloroplast secretion (cpSec) and chloroplast twin-arginine translocation (cpTat) pathways for thylakoid lumen proteins, the chloroplast signal recognition particle (cpSRP)-dependent pathway, the chloroplast spontaneous pathway, and the chloroplast Guided Entry of tail-anchored proteins (cpGET) pathway for thylakoid membrane proteins (Jarvis and Robinson, 2004; Zhu et al., 2022). In general, thylakoid lumen proteins distinctly use the cpSec (when unfolded) and cpTat (when folded) pathways. Moreover, they also have different types of luminal targeting peptides that are cleaved by specific thylakoidal processing peptidases (TPP) (Palmer and Stansfeld, 2020; Xu et al., 2021). Some thylakoid membrane proteins, such as light-harvesting chlorophyll *a/b*-binding proteins (LHCBs), use the cpSRP pathway, which requires a stromal protein complex and a thylakoid membrane-bound integrase. Many proteins, including photosynthetic subunits such as PsaK and PsbW are spontaneously targeted without requiring additional proteins or energy input (Mant et al., 2001; Woolhead et al., 2001; Jarvis and Robinson, 2004; Xu et al., 2021). Recent research has suggested that *Arabidopsis* Get3b can recognize tail-anchored cargo proteins, which have a single transmembrane (TM) domain in their C-terminus followed by a tail of 30 amino acids, and help to deliver them into the thylakoid membrane *via* the cpGET pathway (Anderson et al., 2021; Zhu et al., 2022). In addition, thylakoid membrane proteins generally have different numbers of TM domains in the mature protein region to span the membrane. For example, there are three TM domains in LHCB1, a protein linked to the cpSRP pathway, two TM domains in PsaK, which participates in the spontaneous pathway, and one TM domain in PsbW, which also features a cleavable signaling peptide that is cleaved by TPP inside the thylakoid lumen (Jarvis and Robinson, 2004; Xu et al., 2021; Zhu et al., 2022). This suggests that the specific translocation pathway to be used by a thylakoid membrane-bound protein may depend on the number of TM domain which typically consists of 17-25 hydrophobic amino acids in an α-helix structure (Engelman et al., 1986; Pasquier et al., 1999; White and von Heijne, 2005).

PGs have been recognized as a hub for metabolic activities related to isoprenoid-derived lipids, including phylloquinone, plastoquinone, tocopherols, carotenoids, and apocarotenoids, as well as neutral lipids such as galactolipids, phospholipids, sterol esters, triacylglycerols, and free fatty acids through metabolomic analyses using a diverse array of analytical tools to examine various plastid types (Steinmuller and Tevini, 1985; Vidi et al., 2006; Gaude et al., 2007; van Wijk and Kessler, 2017). Protein profiling has also confirmed the presence of specific metabolic enzymes—including esterases, lipases, and thioesterases—that are involved in the metabolism of neutral lipids. Other enzymes, including ubiE-methyltransferase-related enzymes and ABC1 kinase, which are involved in quinone metabolism, tocopherol cyclase, and carotenoid cleavage dioxygenase 4 have also been identified in PGs in *Arabidopsis* chloroplasts (Vidi et al., 2006; Ytterberg et al., 2006; Rubio et al., 2008; Joyard et al., 2009; Rottet et al., 2016; van Wijk and Kessler, 2017). In addition, one study of saffron found that expression patterns calibrated to the highest levels of β-carotene and β-ionone release were induced during stigma development and that carotenoid cleavage dioxygenase 4 was localized to PGs (Rubio et al., 2008). Another study of *Capsicum* chromoplast PGs confirmed the presence of ζ-carotene desaturase (ZDS), lycopene β-cyclase (*β*-LCY), and β-carotene hydroxylase (CrtR-*β*) during carotenoid biosynthesis (Ytterberg et al., 2006). These results strongly imply that PGs might be the crucial site where lipid metabolites such as isoprenoids (including carotenoids) undergo biosynthesis, degradation, accumulation, and metabolic communication with thylakoids during plant development and environmental adaptation (Brehelin and Kessler, 2008; van Wijk and Kessler, 2017).

Phytoene synthase (PSY), the first and rate-limiting enzyme involved in the carotenoid biosynthesis pathway, has been found to exhibit diverse chloroplast localization, including in the stroma, thylakoid membrane, and PGs, in maize, rice, saffron, and cabbage (Shumskaya et al., 2012; You et al., 2017; Ahrazem et al., 2019; Cao et al., 2021). For example, PG localization of three rice PSYs (OsPSYs) has been reported using a maize protoplast system (Shumskaya et al., 2012). Moreover, our group has previously shown that the OsPSY2 (LOC_Os12g43130; NP_001391692) localizes to PGs in both etiolated and greening rice protoplasts (You et al., 2017). In this study, we predicted that OsPSY2 contains three putative TM helix domains with high hydrophobicity *via* bioinformatics approaches, including querying of four public databases (i.e., the ChloroP 1.1 Server, TMpred, TopPred, and HMMTOP) and the N-terminal 80-amino-acid portion of OsPSY2 (PTp) harbors two putative TM helices with dual localization patterns of fluorescent proteins as cargo, showing speckle-like spots on PGs with a slightly blurred background signal dispersed over the stroma of chloroplasts as a PG-preferential Tp sequence (You et al., 2017). Since the publication of this paper, we hypothesized that the first and second putative TM helix structures in the PTp may be associated with the Tp signal and an additional signal that is partly crucial for PG-targeting of OsPSY2, respectively.

To date, despite there being many cases of PG-targeted proteins, the PG-targeting mechanism has remained largely uncharacterized (Ytterberg et al., 2006; Rubio et al., 2008; Shumskaya et al., 2012; Rottet et al., 2016; You et al., 2017). We determined that OsPSY2 is one of the most promising candidates for identify PG-targeting signals, so we therefore dissected its chloroplast targeting region and membrane spanning domains by generating various combinatory constructs using two fluorescent proteins—i.e., synthetic green fluorescent protein (sGFP) and red fluorescent mCherry protein—as cargo to be delivered. *Via* microscopic fluorescent observation and molecular biological analysis, we elucidate the PG-localization mechanism of OsPSY2 in chloroplasts, and propose strategies for achieving enhanced precision in protein targeting to specific sub-organelle compartments. This precise targeting approach holds great potential for various applications in the field of plant biotechnology.

## Materials and methods

### Vector construction

We previously described the six fluorescent gene fragments such as sGFP (sG), mCherry (mC), a transit peptide of rice RuBisCO small subunit 1 (RTp)-sG, PTp-sG, OsPSY2:mC, and OsPSY2(ΔPTp):mC, which were cloned into *pB2GW7* vector for plant transformation (Dubin et al., 2008; You et al., 2015; You et al., 2017). Using the same system, we first combined RTp and OsPSY2(ΔPTp):mC to generate a RTp-OsPSY2(ΔPTp):mC fragment with overhung recombination sites (attB1/attB2). Next, we PCR-amplified hydrophobic regions (HRs) to obtain new five fragments that contained only one of the following configurations of three hydrophobic helixes (HHs) of OsPSY2. These included HR1, short HR1 (sHR1), HR2, extended HR2 (eHR2), and HR3. Eleven sGFP or mCherry chimeric gene fragments that each included one of these HR fragments and either RTp or PTp are as follows: two for HH1 (no RTp or PTp), one for HH2 (no RTp or PTp), three for HH2 (with RTp), two for HH3 (with RTp), and three for HH3 (with PTp). HR2 was additionally split inside the HH2 structure into front (HR2f) and back (HR2b) halves. Each of these fragments also had overhung attB1/attB2 sites. All PCR reactions were conducted using Phusion^®^ High-Fidelity DNA Polymerase (New England Biolabs, Ipswich, MA, USA) and KOD FX High Success-rate DNA Polymerase (Toyobo, Osaka, Japan). We introduced all twelve gene fragments into a *pB2GW7* vector *via pDONR221* by consecutive recombination using Gateway^®^ BP Clonase^®^ II Enzyme Mix and Gateway^®^ LR Clonase^®^ II Enzyme Mix. All procedures followed the manufacturer’s instructions (Invitrogen, Waltham, MA, USA).

### Rice protoplast isolation and PEG-mediated transfection

Rice (*Oryza sativa* L. Japonica cv. “Ilmi”) seeds were harvested at full maturity (60 days after flowering), were dehusked using a TR-200 Electromotion rice husker (Kett, Tokyo), and were then sterilized by serial treatment of 70% ethanol (once for 1 min), 2% sodium hypochlorite (twice for 20 min), and distilled water (five times). Seeds were then left to germinate on Murashige and Skoog agar medium (Duchefa, Haarlem, Netherlands). Once germinated, they were grown at 28°C for nine days in the dark then for 20 hours under light conditions in a plant growth chamber. Using these seedlings, protoplast isolation was conducted as per our previous description (Bart et al., 2006; You et al., 2015) with minor modifications. In brief, the leaf sheaths of seedlings were cut to 0.5 mm pieces with a sharp razor blade then promptly immersed in a cell wall-degrading enzyme solution (i.e., containing Cellulase R-10 and Macerozyme R-10; Yakult Honsha, Japan) with additional ingredients (i.e., 4 mM CaCl_2_ and 50 µg/mL ampicillin). Plates with rice strips soaked in solution were placed in a desiccation chamber for vacuum infiltration for 10 minutes then incubated with gentle shaking at 50 rpm in the dark at room temperature for 4 hours. This enzyme reaction was stopped by adding three volumes of W5 solution (i.e., 6 mM glucose, 154 mM NaCl, 125 mM CaCl_2_, 10.7 mM KCl, and 2 mM MES adjusted to pH 5.7). After removal of cell debris by filtering twice through 70 and 40 µm cell strainers (SPL, Pocheon, South Korea), rice protoplasts were acquired from the pellet and suspended in MMG buffer (i.e., 0.6 M mannitol, 15 mM MgCl_2_, and 5 mM MES adjusted to pH 5.7) at a concentration of 10^7^ cells/mL.

For transfection, 10^6^ cells were counted using a Marienfeld hemocytometer counting system (Marienfeld-Superior, Berlin, Germany). These aliquots were used for each transfection of plasmid DNA (10 µg) accompanied by an equal volume of 40% PEG-3350 (Sigma, St. Louis, MO, USA) contained in a 0.4 M mannitol and 100 mM Ca(NO_3_)_2_ solution and incubated for 15 minutes. The PEG-mixed protoplasts were then washed twice with two volumes of W5 solution and 1 mL of W5 solution, then resuspended in 1 mL of incubation solution, and incubated at 28°C in the dark overnight. During the transfection experiments, all plastic products were coated with 5% fetal calf serum for 10 seconds and all buffers were filtered using a 0.45µm Sartorius Minisart^®^ syringe filter (Sartorius, Gottingen, Germany).

### Confocal microscope analysis

To observe fluorescence signals indicating subcellular and suborganellar localization in the rice protoplasts, we used Carl Zeiss LSM700 and Carl Zeiss LSM800 inverted confocal microscopes (Carl Zeiss, Oberkochen, Germany). Fluorescence was detected at two excitation and emission wavelengths, i.e., 488 and 509–518 nm for sGFP, 587 and 585–610 nm for mCherry, and 639–680 and 660–700 for chlorophyll autofluorescence, respectively. Fluorescence images were captured using ZEN 2009 Light Edition and ZEN 2.3 Blue Edition software (Carl Zeiss).

### Molecular analysis

The same transfected protoplasts used for fluorescence observations (i.e., approximately 10^6^ cells) were then subjected to protein and RNA extraction. Following microscopy, cells were pelleted, immediately frozen in liquid N_2_, and stored at –80°C until extraction. To extract total protein, rice protoplasts were suspended in 50 μL of 2× Laemmli Sample Buffer (GenDEPOT, Barker, TX, USA), placed in a hot water bath for 10 minutes, then centrifuged at 4°C. Next, 10 μL of the supernatant was loaded onto a 12% acrylamide gel for protein separation *via* SDS-PAGE. Next, the gel was transferred to an Immun-Blot^®^ PVDF Membrane (Bio-Rad) using a Trans-Blot^®^ SD Semi-Dry Transfer Cell (Bio-Rad). Concurrently, a duplicate gel was stained using an EZ-Silver Staining Kit for Protein (Biosesang, Seongnam, Korea) to quantify protein content and estimate the quality of protein extracts. Next, a Western blot was performed using an anti-rabbit polyclonal antibody against GFP (Abcam, Cambridge, UK), an antibody against mCherry (Novus Biologicals, Denver, USA), and an antibody against actin (Abcam, Cambridge, UK). An Anti-Rabbit IgG HRP Conjugate (Promega, Madison, WI, USA) was used as a secondary antibody to react with the primary antibody. Two primary antibodies against GFP and actin were used at dilutions of 1:10,000, and the mCherry antibody was diluted to 1:5000. After incubation with West-Q Femto Clean ECL Solution (GenDEPOT), immunoreactive bands were detected and imaged using an Alliance Mini HD camera (UVITEC, Cambridge, UK).

Total RNA was then extracted from the same rice protoplast samples using RNeasy^®^ Plant mini kits (Qiagen, Hilden, Germany). First strand cDNA was then synthesized using AccuPower^®^ RT Premix (Bioneer, Daejeon, Korea) with all procedures performed according to the manufacturer’s instructions. Quantitative real-time PCR (qRT-PCR) was then performed with the following gene-specific Fwd/Rev primer sets: 5′-GACAAGCAGAAGAACGGCATCA-3′/5′-GGCGGCGGTCACGAACT-3′ for sGFP and 5′-GAAGTAAGGAAGGAGGAGGA-3′/5′-AAGGTGTTCAGTTCCAAGG-3′ for rice ubiquitin (AK061988), which was used as a reference to normalize RNA content. Three technical replicates were performed for each condition. The reaction conditions used for qRT-PCR followed a method that was previously described (You et al., 2017).

## Results

### Generation of recombinant proteins evaluating subcellular localization roles of three hydrophobic regions in OsPSY2

**Figure 1:**
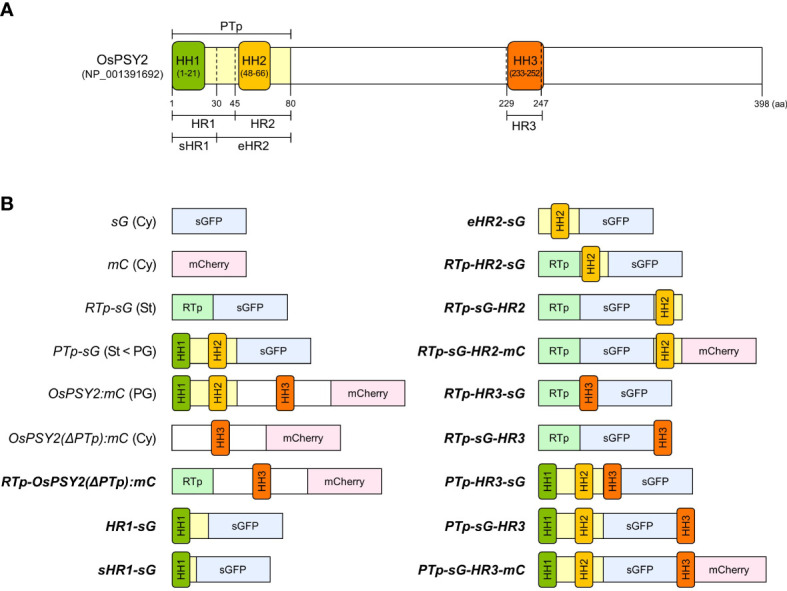
Schematic representation of the OsPSY2 protein with positional information for each region used in this study as well as various fluorescent protein combinations used for rice protoplast transfection. **(A)** OsPSY2 is denoted by the predicted amino acid positions of a transit peptide as predicted by the ChloroP 1.1 Server and hydrophobic regions (HRs) individually containing three hydrophobic helices (HHs) that were identified using the TMpred, TopPred, and HMMTOP databases. **(B)** All chimerically recombined gene fragments including sGFP or/and mCherry are shown in italic for this study. The newly manipulated ones are in boldface (hereafter in all figures). All recombined DNA fragments were cloned into a plant expression vector (i. e., *pB2GW7*) for protein expression in rice protoplasts. OsPSY2, *Oryza sativa* phytoene synthase 2; PTp, the prediction-based N-terminal cleavable transit peptide of OsPSY2; OsPSY2(ΔPTp), OsPSY2 with PTp removed; sHR1, short HR1; eHR2, extended HR2; sG, sGFP; mC, mCherry; RTp, the transit peptide of RuBisCO small subunit 1; Cy, cytosol; St, stroma; PG, plastoglobule.

In a previous study, we reported that the PTp sequence facilitates the simultaneous translocation of foreign proteins into PGs and stroma within chloroplasts. This suggests that there is a need for additional signaling for the intact PG localization of OsPSY2 (You et al., 2017). Based on the predicted TM helix sequences with high hydrophobicity (**Supplementary Figure S1**), we designated three HH structures as HH1 (1–21^st^ aa), HH2 (48–66^th^ aa), and HH3 (233–252^nd^ aa). We also designated the partitioned a PTp (1–80^th^ aa), and two sets of two regions that covered either HH1 or HH2 as follows: ‘HR1 (1–45^th^ aa) and HR2 (46–80^th^ aa)’ and ‘sHR1 (1–30^th^ aa) and eHR2 (31–80^th^ aa)’. Finally, we designated HH3 as ‘HR3 (229–247^th^ aa)’. These residue locations corresponded to a commonly proposed region found in multiple databases (**Figure 1A**). Next, to accurately interpret the results, we used six vectors to express fluorescent proteins such as sGFP or mCherry under the 35S promoter as subcellular and suborganellar markers; these vectors included: sG and mC for the cytosol, RTp-sG for the stroma, PTp-sG for simultaneous but preferring PGs than stroma, OsPSY2:mC for PGs, and OsPSY2(ΔPTp):mC for the cytosol (You et al., 2015; You et al., 2017) (**Figure 1B**). Thus, in this study, we produced a total of fourteen recombinant proteins that should show sGFP or/and mCherry fluorescence; this data helped us to assess whether HH is likely to be a subplastidial targeting signal. We therefore assessed the following possible combinations: two for HH1, four for HH2, three for HH3, and another three for HH3 together with both HH1 and HH2 as the PTp (**Figure 1B**) and additional two for HH2 cut in half front to back (**Supplementary Figure S2A**).

### Chloroplast import property in N-terminal sequences including hydrophobic region 1

**Figure 2:**
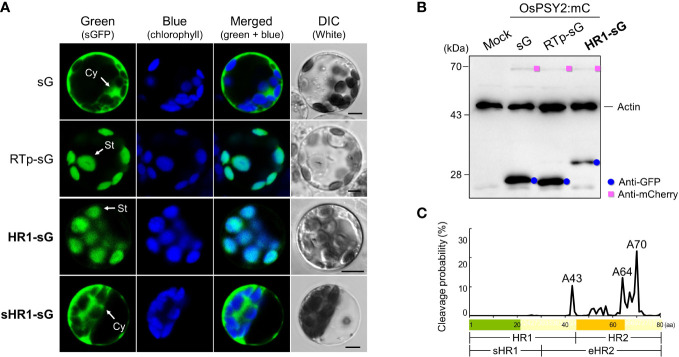
Effect of two N-terminal regions of the OsPSY2 protein including the hydrophobic helix (HH) 1 on subcellular localization in rice protoplasts. **(A)** Individual and merged images of sGFP, chlorophyll, and visible protoplasts using confocal microscopy. sG and RTp-sG were used as a cytosolic (Cy) and stromal (St) localization markers, respectively. Scale bars indicate 5 μm. **(B)** Western blot analysis to compare the amount and size of sGFP. Rice protoplasts were cotransfected with each experimental vector and an OsPSY2:mC vector as a transfection control. This blot was performed with three antibodies simultaneously: Anti-GFP (blue circle), anti-mCherry (pink rectangle), and anti-rice actin (marked at 45 kDa, to be used as an internal reference of the total protein amount). **(C)** Prediction of cleavage probability in the N-terminal 80-amino-acid region of OsPSY2 as implemented by the TargetP 2.0 program (https://services.healthtech.dtu.dk/service.php?TargetP). Estimated cleavage scores of the 80 amino acids of PTp are shown in **Supplementary Table S1**.

Localization of OsPSY2 in PGs within chloroplasts and the fact that its cleaved Tp length is shorter than 80 aa (i.e., as predicted by ChloroP) has previously been examined in maize and rice protoplasts (Shumskaya et al., 2012; You et al., 2017) and pea and rice chloroplasts (Welsch et al., 2008; You et al., 2017), respectively. To determine how OsPSY2 enters the stroma before PGs, two N-terminal regions including HH1 (i.e., HR1 and sHR1) were fused with sGFP and individually transfected into green rice protoplasts from 10-day-old seedlings. Their green fluorescent signals showed different pattern in the stroma and cytosol as matched with the RTp-sG and sG stromal and cytosolic localization markers, respectively (**Figure 2A**). This data therefore suggests that HR1 was responsible for the chloroplast targeting of OsPSY2 and also that the sHR1 lost its function as Tp sequence. This in turn suggests that the 31–45^th^ aa position in the HR1 may be essential for translocation into chloroplasts.

To confirm whether this HR1 was involved in cleavage after translocation into chloroplasts, we performed a Western blot analysis to determine the size of sGFP (**Figure 2B**). The original GFP size of 27 kDa was detected in RTp-sG and sG, whereas HR1-sG generated a much larger GFP band (~32 kDa), suggesting that this is its uncleaved size. We conclude that the HR1 has no cleavable site. To bioinformatically predict the cleavage position of OsPSY2, we then analyzed its protein sequence using the TargetP algorithm (https://services.healthtech.dtu.dk/service.php?TargetP) (Almagro Armenteros et al., 2019). This analysis generated three proposed alanine (A) positions that show high cleavage probability: i.e., A43 in HR1 and A64 and A70 in the HR2 (**Figure 2C** and **Supplementary Table S1**). These data suggest that OsPSY2 is more likely to be truncated in HR2 and not in the HR1, since A43 is experimentally excluded (**Figure 2B**).

### The role of hydrophobic region 2 as a partial PG-targeting signal and for cleavage in chloroplasts

**Figure 3:**
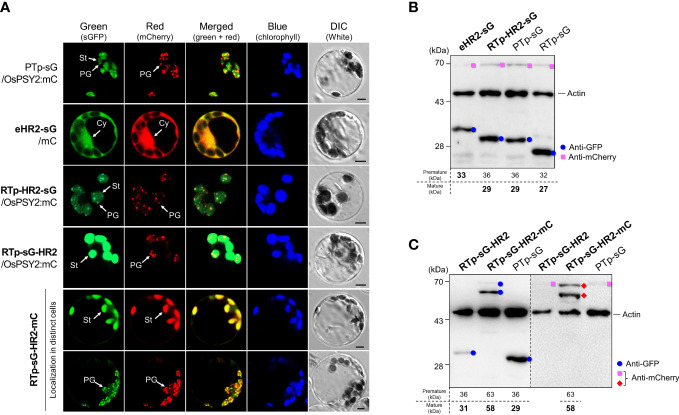
Effect of the hydrophobic region (HR) 2 on subcellular and suborganellar localization in rice protoplasts. **(A)** Individual and merged images of sGFP, mCherry, chlorophyll, and visible protoplasts using confocal microscopy. PTp-sG, OsPSY2:mC, and mC were used as markers for dual localization into plastoglobules (PGs) and stroma (St) as well as single localization into a PG and the cytosol (Cy), respectively. Scale bars indicate 5 μm. **(B)** Western blot analysis to confirm the cleavage in HR2 when placed right after a Tp signal such as RTp or HR1 within PTp. **(C)** Western blot analysis to confirm that no cleavage occurred in HR2 when placed at the C-terminus of sGFP. In both **(B, C)**, rice protoplasts were cotransfected with an experimental vector and an OsPSY2:mC vector used as a transfection control. In one exceptional case we used RTp-sG-HR2-mC with intrinsic mC. The blot shown in **(B)** was performed with three antibodies simultaneously: Anti-GFP (blue circle), anti-mCherry (red diamond for the experimental vector or pink rectangle for the control vector), and anti-rice actin (marked at 45 kDa and used as an internal reference indicating total protein amount). The blot shown in **(C)** was performed with two antibodies simultaneously: Anti-GFP and anti-rice actin in the left panel, and anti-mCherry and anti-rice actin in the right panel, respectively. The expected protein size is noted under the blot.

To acertain the influence of HH2 on the translocation of OsPSY2, we cotransfected green rice protoplasts with PTp-sG or one of three new recombinant fluorescent proteins harboring regions containing HH2 with either mC or OsPSY2:mC; we also transfected protoplasts with RTp-sG-HR2-mC alone (**Figure 3A**). The eHR2 resulted in cytosolic localization, confirming that it alone was not functional as Tp despite containing HH2 and a 30-aa N-terminal region including HH1 (i.e., sHR1) might necessitate first entry into chloroplasts again. To understand the unique features of putative HR2 in PTp, we used RTp instead of HR1 and placed HR2 both before and after sG, thus generating both RTp-HR2-sG and RTp-sG-HR2. The green fluorescence signal of RTp-HR2-sG spread throughout the stroma in discrete specks. This punctuate pattern exactly overlapped with the red PG signals of OsPSY2:mC when cotransfected. The overall dual targeting pattern of RTp-HR2-sG (i.e., including both the stroma and PGs) was similar to PTp-sG, providing evidence that HR2 may be involved in PG-targeting. In contrast, the green fluorescence signal of RTp-sG-HR2 was displayed only in the stroma, indicating that HR2 placement is dependent on RTp. To verify this finding, we generated an additional collocation of mC in the C-terminus to form RTp-sG-HR2-mC. We then observed two asynchronous patterns of either stroma or PGs in individual cells. Interestingly, we found that when RTp (used as a substitute of HR1) and HR2 were close and not far, as in RTp-HR2-sG and PTp-sG, they exerted the dual-target pattern. However, when they were spaced further apart, as in RTp-sG-HR2 and RTp-sG-HR2-mC, they displayed a single-target pattern. This suggests that RTp/HR1 and HR2 may be in competition and also that the position of HR2 in cargo proteins works crucially as a PG-targeting signal: i.e., at the N-terminus as in RTp-HR2-sG and PTp-sG, in the middle as in RTp-sG-HR2-mC, but never at the C-terminus as in RTp-sG-HR2.

To determine whether and where cleavage occurs for OsPSY2, we conducted Western blots using rice protoplasts and compared the sizes of fluorescent proteins produced (**Figure 3B**). These results showed that the uncleaved GFP band of eHR2-sG was coincident with a failure to target chloroplasts. Moreover, it also showed the same cleaved size GFP bands from RTp-HR2-sG and PTp-sG were greater than the 28 kDa protein marker as well as the mature GFP produced by RTp-sG. This proves that the cleavage occurred in HR2 in both RTp-HR2-sG and PTp-sG and that the mature GFP size was equivalent to the theoretical size following cleavage at the A64 position. This means that the estimated size is either 29 or 28 kDa if cleaved at either A64 or A70 in HR2. Moreover, the fact that RTp-sG-HR2 displayed a bigger GFP band than PTp-sG (which was equivalent to RTp-HR2-sG) confirmed that RTp-HR2-sG had a second cleavage in HR2 following the first cleavage of RTp. Thus, cleavage occurred once in the HR2 of PTp-sG, twice in RTp and the HR2 of RTp-HR2-sG, and once in RTp of RTp-sG-HR2 and RTp-sG-HR2-mC (**Figure 3C**).

To further verify which region is involved in PG-targeting and whether cleavage actually occurs at the A64 position, a HR2 was divided into two of a 21-aa in the front (HR2f, 46-66^th^ aa) and a 17-aa on the back (HR2b, 64-80^th^ aa) and recombined them with RTp and sGFP in the N- and C-terminus, respectively (**Supplementary Figures S2A, B**). The cleavage positions of RTp-linked protein sequences were commonly predicted the probability of A64 in HR2 with cysteine (C) 48 in RTp region (**Supplementary Figure S2C**). The subplastidial localization showed different fluorescent patterns between RTp-HR2f-sG and RTp-HR2b-sG: the former in only stroma and the latter in PGs and stroma with similarity to PTp-sG, confirming that PG-targeting ability of a PTp might be due to the 16 aa-region on the back of HR2 after cleavage at the A64 (**Supplementary Figure S2D**). Western blot also confirmed the occurrence of actual cleavage within HR2f showing smaller GFP band in RTp-HR2f-sG (27 kDa) than RTp-HR2-sG (29 kDa) as predictably cleaved sizes at the A64 (**Supplementary Figure S2E**).

Taken together, our results indicate that HR2 containing HH2 requires a front-facing domain containing another HH structure, such as RTp or HR1 of PTp, for plastidial cleavage and chloroplast import. With respect to PG localization, HR2 can play a part unless it is positioned at the C-terminus of a plastidial cargo protein.

### Role of hydrophobic region 3 as a novel PG-targeting signal requiring a precise location

**Figure 4:**
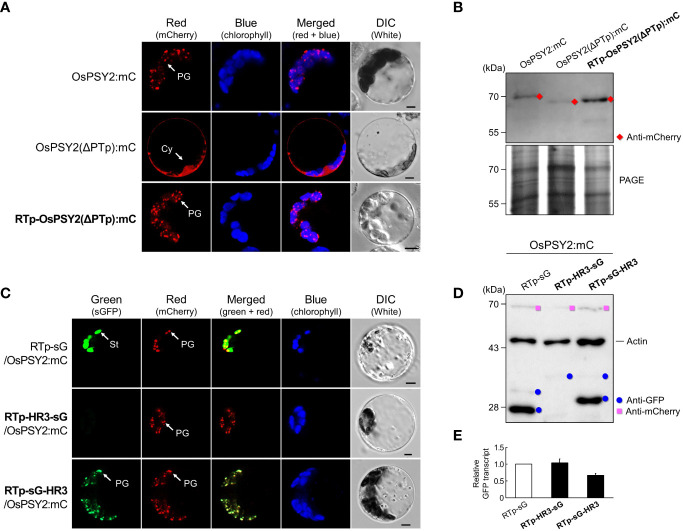
Effect of the hydrophobic region (HR) 3 on PG localization in rice protoplasts. **(A)** Individual and merged images of mCherry, chlorophyll, and visible protoplasts using confocal microscopy. OsPSY2:mC and OsPSY2(ΔPTp):mC were used as localization markers of plastoglobules (PGs) and the cytosol (Cy), respectively. Scale bars indicate 5 μm. **(B)** Western blot analysis to confirm the presence of a PG-targeting signal in an area other than PTp in OsPSY2. Rice protoplasts were individually transfected with three experimental vectors. This blot was conducted with anti-mCherry (red diamond) and a polyacrylamide gel electrophoresis (PAGE) image is shown to indicate total protein amounts. **(C)** Individual and merged images of sGFP, mCherry, chlorophyll, and visible protoplasts using confocal microscopy. RTp-sG and OsPSY2:mC were used as localization markers for the stroma (St) and plastoglobules (PGs), respectively. Scale bars indicate 5 μm. **(D)** Western blot analysis to reveal the precise location of HR3 that exhibits PG-targeting capacity. Rice protoplasts were cotransfected with an experimental vector and an OsPSY2:mC vector, used as a transfection control. The blot was performed with three antibodies simultaneously: Anti-GFP (blue circle), anti-mCherry (pink rectangle), and anti-rice actin (marked at 45 kDa and used as an internal reference of total protein amount). **(E)** Quantitative real-time-PCR to indicate relative transcript levels depending on different protein levels for the three vectors. The rice ubiquitin gene (AK061988) was used as a reference to normalize RNA amounts. qRT-PCR was performed using three technical replicates with three biological samples per vector.

In a previous study, we hypothesized that a signal other than PTp may be required for complete PG targeting, since there was a discrepancy in subplastidial localization of PTp, which was simultaneously localized to PGs and the stroma, and OsPSY2, which localized only to PGs (You et al., 2017). To confirm the presence of this signal, we prepared a new RTp-OsPSY2(ΔPTp):mC and compared its localization pattern to OsPSY2:mC and OsPSY2(ΔPTp):mC (**Figure 4A**). We found that the red fluorescence signal of RTp-OsPSY2(ΔPTp):mC showed the same PG-localization pattern of OsPSY2:mC, confirming our assumption that there is another PG-targeting signal in OsPSY2. In addition, the RTp-cleaved mature protein was also confirmed as having the same size as the cytosolic-targeted OsPSY2(ΔPTp):mC (**Figure 4B**).

At this point, we speculated whether HH3 works as a novel PG-signal located in the mature region of OsPSY2. Therefore, we contrived two recombinant proteins to collocate HR3 before and after sG, obtaining RTp-HR3-sG and RTp-sG-HR3. In rice protoplasts, RTp-sG-HR3 displayed a PG-targeting pattern, which was consistent with that of the cotransfected OsPSY2:mC (i.e., showing specks dotted within chloroplasts). This pattern was totally different than RTp-sG, which showed stromal localization, as well as RTp-HR3-sG, which had no fluorescent signal (**Figure 4C**). To further analyze these proteins on the molecular level, we examined protein production *via* Western blot (**Figure 4D**). Compared to the cleaved 27 kDa GFP band of RTp-sG, RTp-sG-HR3 produced a cleaved 29 kDa GFP band but RTp-HR3-sG formed only a weak premature GFP band. Additional quantitative real-time-PCR was performed to examine whether there were differences in transcript levels (**Figure 4E**). Interestingly, all three showed similar levels of normal GFP transcription despite different patterns of fluorescence and protein expression. These findings suggest that HR3 causes no protein production if located at the N-terminus of a cargo protein, where it is placed side-by-side with RTp, but also that HR3 alone can exert a near-perfect PG-targeting role if located at the C-terminus of a chloroplastidial protein when translocated by a simple Tp such as RTp.

### The mode of hydrophobic region 3 action for PG localization under the influence of hydrophobic region 1 and 2

**Figure 5:**
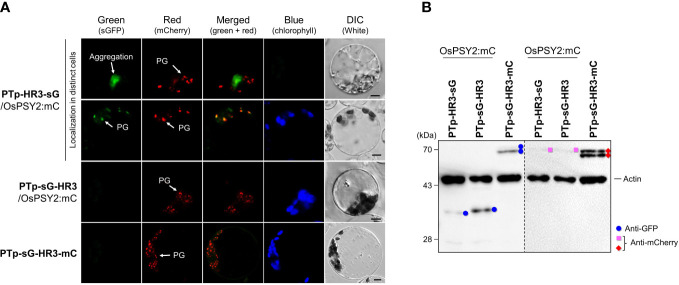
Differential effects of the hydrophobic region (HR) 3 on the PG-localization depending on PTp in rice protoplasts. **(A)** Individual and merged images of sGFP, mCherry, chlorophyll, and visible protoplasts using confocal microscopy. OsPSY2:mC was used as a plastoglobule (PG) localization marker. Scale bars indicate 5 μm. **(B)** Western blot analysis to determine the factors influencing aggregation and loss of fluorescence. Rice protoplasts were cotransfected with an experimental vector and an OsPSY2:mC vector used as a transfection control. In one exceptional case PTp-sG-HR3-mC was used, which has intrinsic mC. The blot was performed with two antibodies simultaneously: Anti-GFP (blue circle) and anti-rice actin (marked at 45 kDa) on the left panel, and anti-mCherry (red diamond for experimental vector or pink rectangle for the control vector) and anti-rice actin on the right panel, respectively.

In addition to the PG-targeting role of HR3 in collaboration with RTp, we also examined the characteristics of HR3 when towed by PTp. To do so, we transfected rice protoplasts with three recombinant proteins that included HR3 in different places around fluorescent cargo proteins: i.e., the N-terminus of sG, the C-terminus of sG, and in between sG and mC (**Figure 5A**). In the PTp-HR3-sG case, we identified two different types of green fluorescence, either aggregation or (rarely) a PG-targeting pattern. This matched the pattern of cotransfected OsPSY2-mC. At the same time, PTp-sG-HR3 did not show any fluorescent signal. Taken together, these data suggest that the close arrangement of three HHs in a row easily forms fluorescent aggregates and also that the ‘trapped’ pattern in which sGFP is between two HHs disturbs fluorescent expression. Next, we tried to reproduce the configuration of OsPSY2 *via* mC-fusion at C-terminus of PTp-sG-HR3, generating PTp-sG-HR3-mC. Observation of its red fluorescent signal revealed PG localization. This presented evidence that HR3 has a PG-targeting ability and requires a precise location to be functional.

To further understand the protein dynamics at play, we performed subsequent Western blots and observed that the fluorescent proteins corresponding to sG and mC (e.g., PTp-sG-HR3-mC) were normally produced at the mature size (**Figure 5B**). This finding revealed that the barely observed fluorescent green signals were caused by posttranslational effects; e.g., clumping that caused relatively lower amounts of GFP production for PTp-HR3-sG and folding errors despite normal GFP production for PTp-sG-HR3 and PTp-sG-HR3-mC. In addition, the mC contained in PTp-sG-HR3-mC showed successful PG-targeted red fluorescence with normal protein expression. It implies that the HR3 position does not affect PG targeting itself but that there must be distance between HR2 and HR3 and that HR3 must not be positioned at the C-terminus of a cargo protein when translocated by PTp containing HR1 and HR2.

## Discussion

Since the reports of “osmiophilic globules” in faba bean, spinach, and beet chloroplasts (Bailey and Whyborn, 1963; Greenwood et al., 1963), PGs have been identified as active subcompartments. That is, rather than passive storage droplets for lipid metabolism including carotenogenesis, they engage in two-way communication with the thylakoid membrane (Austin et al., 2006; Brehelin and Kessler, 2008; Zita et al., 2022). In addition that the presence of carotenoid biosynthetic enzymes such as ZDS, *β*-LCY, and CrtR-*β* with high levels of ketoxanthophylls such as capsanthin and capsorubin has been found in the chromoplast PGs of red bell pepper (Ytterberg et al., 2006; van Wijk and Kessler, 2017), the enriched accumulation of carotenogenic enzymes such as PSY (i.e., by 7.5-fold), phytoene desaturase (7.2-fold), carotenoid isomerase (12-fold) was reported alongside large increases of lycopene and *β*-carotene in chromoplasts relative to chloroplasts in the PG proteome and metabolome of tomato (Zita et al., 2022). These results strongly support the notion that the PG-localized carotenoid biosynthetic enzymes, which includes PSY, might directly contribute to carotenoid biosynthesis. Previously, Shumskaya et al. (2012) and our group provide evidence of the PG localization of OsPSY2 (You et al., 2017) and we suggested that the prediction-based Tp region of OsPSY2 drove the dual localization of this protein to PGs and the stroma. This finding left room for further study to elucidate PG-targeting signals in areas other than PTp (**Figure 1A**).

**Figure 6:**
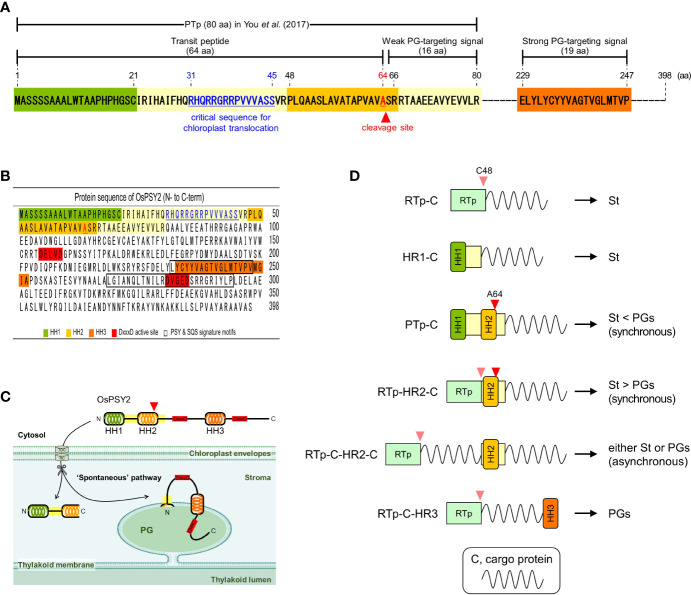
Characteristics of OsPSY2 destined for plastoglobuli localization and potential strategies for subplastidial localization. **(A)** Summary of features identified as functioning as a transit peptide and PG-targeting signal in this study. **(B)** Protein sequence information of OsPSY2, including important regions (marked in colors) including the hydrophobic helix (HH) sequences. **(C)** Proposed model indicating how OsPSY2 exists in PGs within plastids. **(D)** Proposal of six strategies for delivery of proteins of interest into the stroma and PGs with different preferences. All abbreviations here refer to **Figures 1A, B**.

In this study, we generated a variety of recombinant proteins with stepwise combinations of three HRs of OsPSY2 (**Figure 1B**). Using these recombinant proteins, we investigated protein fluorescence and expression in rice protoplast systems. First, we identified the critical region for chloroplast targeting as a 15-aa-peptide in the region between residues 31–45 located between HH1 and HH2 (**Figures 2A, B**). We also identified cleavage site A64 positioned at the end of HH2 (**Figures 2C** and **3B**). Next, we determined that the factual Tp of OsPSY2 is a 64-aa-long protein including HH1 and most of HH2; this is compared to the 80-aa-long sequence of PTp identified *via* program-driven prediction (**Figure 6A**). After translocation into a chloroplast, OsPSY2 might undergo a secondary translocation into PGs. To understand this mechanism, HR2 and HR3 were individually relocated at either the N- or C-terminus of sGFP and in a middle location between sGFP and mCherry at a position after stroma cleavage (**Figure 1B**). We attested the weak PG-targeting capacity of HR2 to positional effects caused by different influences on HR1 and RTp that result in three patterns of dual localization into PG (by HR2) and the stroma (by HR1 or RTp). These patterns were biased toward PGs for PTp-sG, toward the stroma for RTp-HR2-sG and was balanced for RTp-sG-HR2-mC (**Figure 3A**). Here we determined that for HR2, most of HH2 is cut out by stromal processing at A64; the remaining 16-aa-region hangs at the N-terminus of the mature protein. The possibility that this region may be more influential than HH2 itself as a PG-targeting signal was also verified (**Supplementary Figure S2**). In addition, the capacity of HR3 to drag cargo proteins into PGs is evidently greater than HR2; this conclusion is supported by our observation of clear PG-targeting patterns RTp-sG-HR3 (visualized using GFP) and PTp-sG-HR3-mC (visualized using mCherry), respectively (**Figure 4C** and **5A**). Taken together, we identified all the features in OsPSY2 to act as a transit peptide, including the critical region for translocation and the cleavage site and features related to weak and strong PG-targeting signals (**Figure 6A**).

Interestingly, for HR3 we observed diverse aberrant patterns of GFP fluorescence for RTp-HR3-sG, PTp-HR3-sG, PTp-sG-HR3, and PTp-sG-HR3-mC. The first case showed insufficient protein translation (not transcription), but the other three showed different posttranslational errors. This causes aggregates to form in the second case and folding errors to occur in the third and fourth cases (**Figures 4D** and **5B**). Thus, no protein detected for RTp-HR3-sG might be caused by the elimination of unfolded, misfolded, or unimported proteins for protein homeostasis as part of general cell clean-up routines (Sun et al., 2021). This hypothesis is supported by our observation of a very thin, premature size of the GFP band (**Figure 4D**). This revealed the strong demand of HR3 for an exact location to preserve proper protein function. Thus, it requires sufficient distance from any other HH structure and must not be located at the C-terminal end of a cargo protein that also contains an N-terminal HH.

In general, PGs are born in highly curved margins of thylakoid membranes by blistering of the monolayer (Austin et al., 2006). It implies that PG-targeting mechanisms have a high probability of following a similar translocation path as do thylakoid membrane proteins: i.e., *via* one of the cpSRP, spontaneous, and/or cpGET pathways (Xu et al., 2021; Zhu et al., 2022). In that respect, the spontaneous pathway, which was previously reported for PsaK (Jarvis and Robinson, 2004), is the most plausible as the PG-translocation path of OsPSY2 since it has both HR2 and HR3 as well as HR1. By considering the overall protein structure, including the regions in which OsPSY2 shows activity (**Figure 6B**), we propose a model of how OsPSY2 is embedded in the PG-monolayer by two membrane-penetrating parts. This results in exposure of the first on the stromal side and the second inside the PG-monolayer among two DxxxD active sites (**Figure 6C**).

So far, the best known Tp is a 50-aa-long-RTp which has a predicted HH (1–20^th^ aa) and is known to be cleaved at C48 by stromal processing (Eseverri et al., 2020). Here, we used our results to strategize for achieving subplastidial locations between PGs and the stroma (**Figure 6D**). The six strategies identified here may be useful for the delivery of cargo proteins into micro-organelle compartments, where they can work best for key plant biotechnology applications such as metabolic engineering, protein engineering, and molecular farming.

## Data availability statement

The original contributions presented in the study are included in the article/**Supplementary Material**. Further inquiries can be directed to the corresponding author.

## Author contributions

JY performed all experiments, including bioinformatics, vector cloning, subcellular localization, and molecular analysis, and wrote the draft. MY contributed to the experimental design and data interpretation and wrote the draft with JY. YL contributed to rice protoplast preparation and confocal microscopic analysis. S-HH envisioned and coordinated the project, supervised the manuscript, and is responsible for all contacts and correspondence. All authors contributed to the article and approved the submitted version.

## Supplementary material

Supplementary Figure 1 Prediction of hydrophobic helix (HH) structures in OsPSY2. **(A)** Hydrophobic degree to propose the potent hydrophobic alpha helix structures in protein sequences of OsPSY2 that was analyzed using the TMpred server. Three putative hydrophobic amino acid regions are plotted as solid lines above the peaks. **(B)** Hydrophobic residue distribution of three HH structures of OsPSY2. The number on both sides indicates the amino acid residue position of the OsPSY2. The hydrophobic residues that are generally insoluble and uncharged in aqueous solution (i. e., A, F, I, L, M, P, V, W, and Y), are marked in red letters.

Supplementary Figure 2 Verification of PG-targeting signal and cleavage occurrence at the A64 position inside the hydrophobic region (HR) 2 in rice protoplasts. **(A)** Schematic representation showing three recombinant genes of rice RuBisCO small subunit 1 transit peptide (RTp) and each of three different HR2 sequences to express green fluorescent proteins. All DNA fragments in italic were cloned into a plant expression vector (i. e., *pB2GW7*). **(B)** Protein sequence information in a full-length of HR2, a front half-length of HR2 (HR2f), and a back half-length of HR2 (HR2b) together with the simplified protein sequence of RTp. The known and predicted cleavable sites in **(C)** were bold and underlined with triangle marks. **(C)** Prediction results of cleavage probability of three RTp-fused protein regions with different HR2 parts, respectively, by the TargetP 2.0 program. **(D)** Individual and merged images of sGFP, mCherry, chlorophyll, and differential interference contrast (DIC) picture of rice protoplasts using confocal microscopy. OsPSY2:mC was used as a marker for PG localization. **(E)** Western blot analysis to confirm whether cleavage is occurring at A64 position inside HR2. Rice protoplasts were cotransfected with each experimental vector and an OsPSY2:mC vector as a transfection control. This blot was performed with three antibodies simultaneously: anti-GFP (blue circle), anti-mCherry (pink rectangle), and anti-rice actin (marked at 45 kDa, to be used as an internal reference of the total protein amount).
