# Supplementary material for: Stepwise protein targeting into plastoglobules are facilitated by three hydrophobic regions of rice phytoene synthase 2
Source: Front Plant Sci. 2023 May 31;14:1181311. doi: 10.3389/fpls.2023.1181311 (PMC10264786; doi:10.3389/fpls.2023.1181311)
Supplement: Supplementary Figure 1 — Prediction of hydrophobic helix (HH) structures in OsPSY2. (A) Hydrophobic degree to propose the potent hydrophobic alpha helix structures in protein sequences of OsPSY2 that was analyzed using the TMpred server. Three putative hydrophobic amino acid regions are plotted as solid lines above the peaks. (B) Hydrophobic residue distribution of three HH structures of OsPSY2. The number on both sides indicates the amino acid residue position of the OsPSY2. The hydrophobic residues that are generally insoluble and uncharged in aqueous solution (i. e., A, F, I, L, M, P, V, W, and Y), are marked in red letters. [file Presentation_1.pptx]

## Slide 1
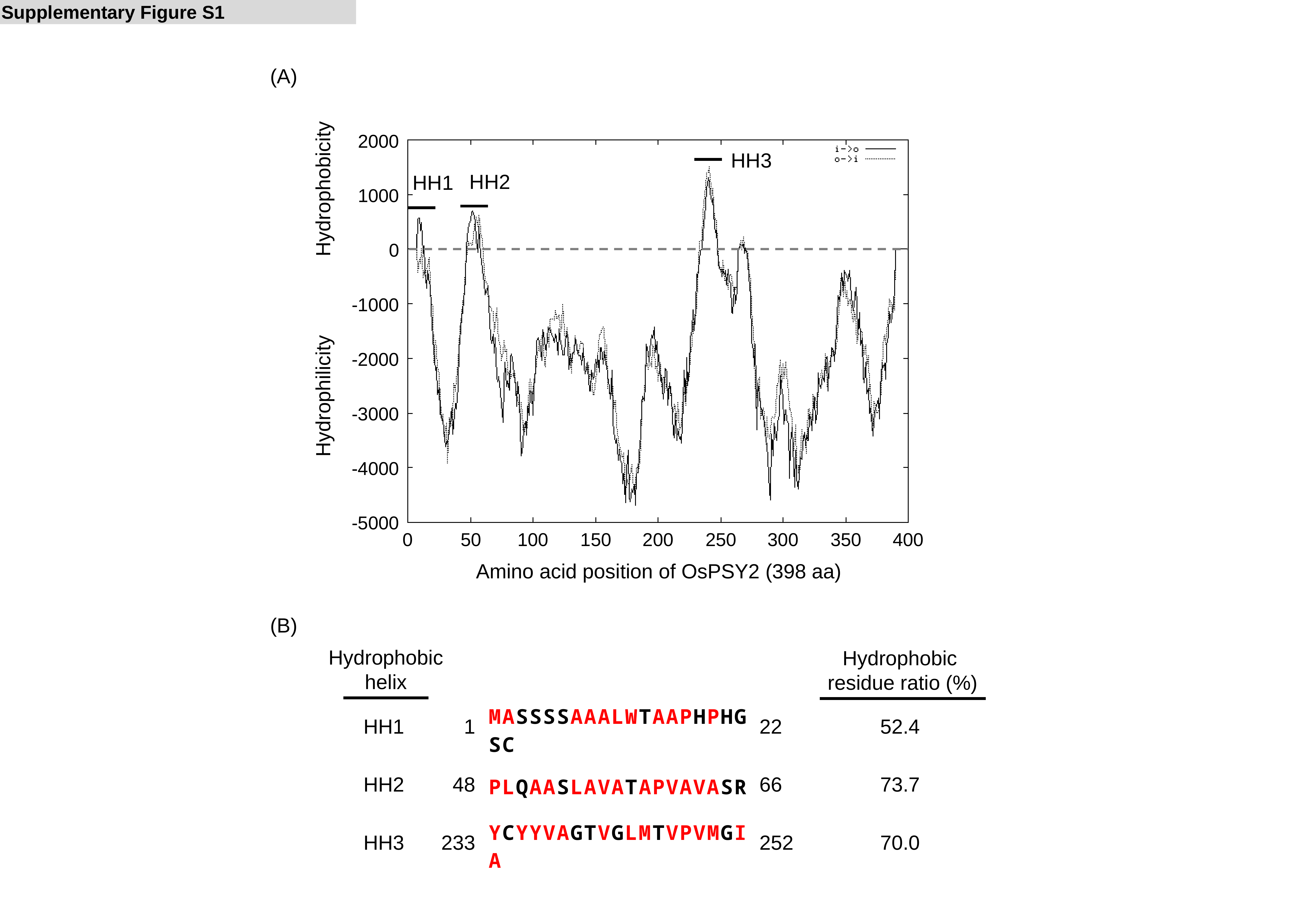

Supplementary Figure S1
(A)
2000
HH3
HH2
HH1
Hydrophobicity
1000
0
-1000
-2000
Hydrophilicity
-3000
-4000
-5000
0
50
100
150
200
250
300
350
400
Amino acid position of OsPSY2 (398 aa)
(B)
Hydrophobic
helix
Hydrophobic
residue ratio (%)
| HH1 | 1 | MASSSSAAALWTAAPHPHGSC | 22 | 52.4 |
| --- | --- | --- | --- | --- |
| | | | | |
| HH2 | 48 | PLQAASLAVATAPVAVASR | 66 | 73.7 |
| | | | | |
| HH3 | 233 | YCYYVAGTVGLMTVPVMGIA | 252 | 70.0 |

## Slide 2
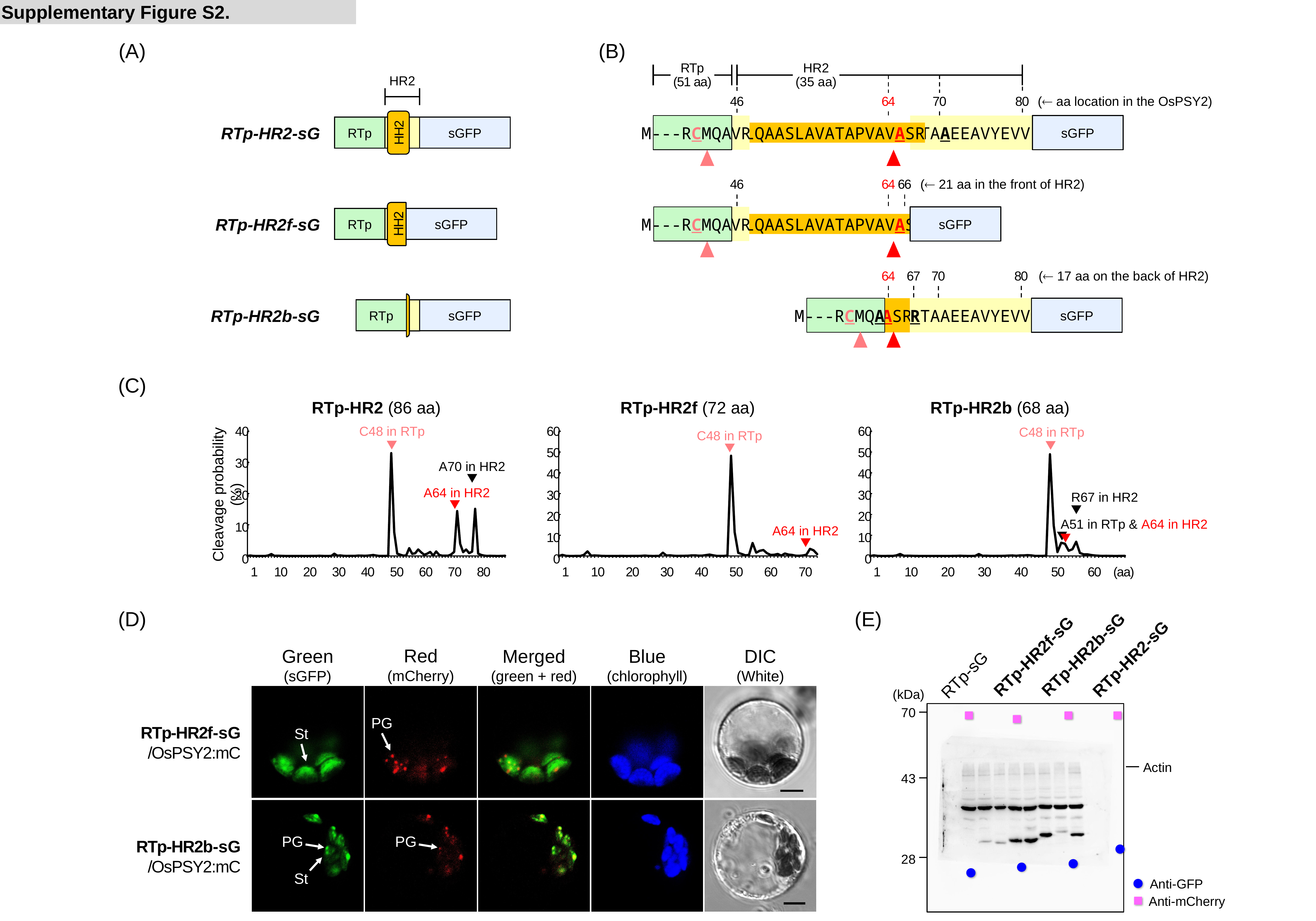

Supplementary Figure S2.
(A)
(B)
RTp
(51 aa)
HR2
(35 aa)
HR2
RTp
sGFP
RTp-HR2-sG
HH2
RTp
sGFP
RTp-HR2f-sG
RTp
sGFP
RTp-HR2b-sG
46
64
70
80
( aa location in the OsPSY2)
sGFP
M---RCMQA
VR
PLQAASLAVATAPVAVASR
RTAAEEAVYEVVLR
46
64
66
( 21 aa in the front of HR2)
sGFP
M---RCMQA
VR
PLQAASLAVATAPVAVASR
64
67
70
80
( 17 aa on the back of HR2)
sGFP
RTAAEEAVYEVVLR
M---RCMQA
ASR
(C)
RTp-HR2 (86 aa)
RTp-HR2f (72 aa)
RTp-HR2b (68 aa)
40
60
60
C48 in RTp
C48 in RTp
### Chart
| Category | |
|---|---|
| 0 | 0.0 |
| 1 | 0.146 |
| 2 | 0.0161 |
| 3 | 0.0009 |
| 4 | 0.0028 |
| 5 | 0.0078 |
| 6 | 0.0055000000000000005 |
| 7 | 0.1802 |
| 8 | 0.6152000000000001 |
| 9 | 0.056499999999999995 |
| 10 | 0.0783 |
| 11 | 0.057499999999999996 |
| 12 | 0.0127 |
| 13 | 0.0036 |
| 14 | 0.0021 |
| 15 | 0.0034 |
| 16 | 0.0025 |
| 17 | 0.0063999999999999994 |
| 18 | 0.0085 |
| 19 | 0.0034 |
| 20 | 0.0052 |
| 21 | 0.0086 |
| 22 | 0.0085 |
| 23 | 0.0232 |
| 24 | 0.0736 |
| 25 | 0.015899999999999997 |
| 26 | 0.0021 |
| 27 | 0.0043 |
| 28 | 0.015799999999999998 |
| 29 | 0.7246 |
| 30 | 0.1089 |
| 31 | 0.17179999999999998 |
| 32 | 0.061 |
| 33 | 0.0174 |
| 34 | 0.035500000000000004 |
| 35 | 0.0366 |
| 36 | 0.0446 |
| 37 | 0.11839999999999999 |
| 38 | 0.11050000000000001 |
| 39 | 0.0616 |
| 40 | 0.0893 |
| 41 | 0.1976 |
| 42 | 0.3191 |
| 43 | 0.1414 |
| 44 | 0.0162 |
| 45 | 0.0115 |
| 46 | 0.013 |
| 47 | 0.0692 |
| 48 | 32.9916 |
| 49 | 7.5946 |
| 50 | 0.7411 |
| 51 | 0.4158 |
| 52 | 0.1178 |
| 53 | 0.1745 |
| 54 | 2.4962999999999997 |
| 55 | 0.6913 |
| 56 | 0.8649 |
| 57 | 2.0902 |
| 58 | 1.1699 |
| 59 | 0.3912 |
| 60 | 0.7342 |
| 61 | 1.3025 |
| 62 | 0.127 |
| 63 | 1.4460000000000002 |
| 64 | 0.3401 |
| 65 | 0.0967 |
| 66 | 0.0858 |
| 67 | 0.0998 |
| 68 | 0.4952 |
| 69 | 1.3161 |
| 70 | 14.407300000000001 |
| 71 | 3.9114999999999998 |
| 72 | 1.2293 |
| 73 | 2.0872 |
| 74 | 0.9112 |
| 75 | 1.4091 |
| 76 | 15.132599999999998 |
| 77 | 0.7451 |
| 78 | 0.3463 |
| 79 | 0.1117 |
| 80 | 0.019799999999999998 |
| 81 | 0.056400000000000006 |
| 82 | 0.026699999999999998 |
| 83 | 0.004699999999999999 |
| 84 | 0.004699999999999999 |
| 85 | 0.022699999999999998 |
| 86 | 0.076 |
### Chart
| Category | |
|---|---|
| 0 | 0.0 |
| 1 | 0.5229 |
| 2 | 0.0807 |
| 3 | 0.0038000000000000004 |
| 4 | 0.01 |
| 5 | 0.0246 |
| 6 | 0.0229 |
| 7 | 0.5412 |
| 8 | 2.2182 |
| 9 | 0.1686 |
| 10 | 0.2345 |
| 11 | 0.1753 |
| 12 | 0.038 |
| 13 | 0.0118 |
| 14 | 0.0103 |
| 15 | 0.0112 |
| 16 | 0.009399999999999999 |
| 17 | 0.0168 |
| 18 | 0.0169 |
| 19 | 0.009399999999999999 |
| 20 | 0.0145 |
| 21 | 0.0201 |
| 22 | 0.0237 |
| 23 | 0.06989999999999999 |
| 24 | 0.181 |
| 25 | 0.0536 |
| 26 | 0.0068 |
| 27 | 0.013300000000000001 |
| 28 | 0.039 |
| 29 | 1.5744999999999998 |
| 30 | 0.22390000000000002 |
| 31 | 0.3025 |
| 32 | 0.1087 |
| 33 | 0.0412 |
| 34 | 0.08009999999999999 |
| 35 | 0.0903 |
| 36 | 0.1119 |
| 37 | 0.2697 |
| 38 | 0.28400000000000003 |
| 39 | 0.1353 |
| 40 | 0.1691 |
| 41 | 0.42389999999999994 |
| 42 | 0.6747000000000001 |
| 43 | 0.328 |
| 44 | 0.0423 |
| 45 | 0.0404 |
| 46 | 0.043199999999999995 |
| 47 | 0.1943 |
| 48 | 48.2443 |
| 49 | 11.4946 |
| 50 | 1.508 |
| 51 | 0.9483999999999999 |
| 52 | 0.3155 |
| 53 | 0.5139 |
| 54 | 6.2399000000000004 |
| 55 | 1.6125 |
| 56 | 2.4988 |
| 57 | 2.8644 |
| 58 | 1.3576 |
| 59 | 0.5218999999999999 |
| 60 | 0.5635 |
| 61 | 0.9802 |
| 62 | 0.1751 |
| 63 | 1.1868 |
| 64 | 0.6753 |
| 65 | 0.5073 |
| 66 | 0.12719999999999998 |
| 67 | 0.0941 |
| 68 | 0.2464 |
| 69 | 0.6192 |
| 70 | 3.4356999999999998 |
| 71 | 2.7093 |
| 72 | 0.8743000000000001 |
### Chart
| Category | |
|---|---|
| 0 | 0.0 |
| 1 | 0.29009999999999997 |
| 2 | 0.045 |
| 3 | 0.0028 |
| 4 | 0.007600000000000001 |
| 5 | 0.0205 |
| 6 | 0.0151 |
| 7 | 0.2777 |
| 8 | 0.9594999999999999 |
| 9 | 0.0968 |
| 10 | 0.12459999999999999 |
| 11 | 0.09380000000000001 |
| 12 | 0.0265 |
| 13 | 0.0086 |
| 14 | 0.0053 |
| 15 | 0.0069 |
| 16 | 0.0055000000000000005 |
| 17 | 0.0091 |
| 18 | 0.0081 |
| 19 | 0.0057 |
| 20 | 0.0085 |
| 21 | 0.01 |
| 22 | 0.0138 |
| 23 | 0.0452 |
| 24 | 0.11499999999999999 |
| 25 | 0.0381 |
| 26 | 0.0043 |
| 27 | 0.0073999999999999995 |
| 28 | 0.026400000000000003 |
| 29 | 0.9238 |
| 30 | 0.0881 |
| 31 | 0.1156 |
| 32 | 0.0518 |
| 33 | 0.0265 |
| 34 | 0.0494 |
| 35 | 0.0594 |
| 36 | 0.0853 |
| 37 | 0.1845 |
| 38 | 0.23909999999999998 |
| 39 | 0.0902 |
| 40 | 0.25630000000000003 |
| 41 | 0.2678 |
| 42 | 0.39880000000000004 |
| 43 | 0.2792 |
| 44 | 0.041800000000000004 |
| 45 | 0.0274 |
| 46 | 0.033 |
| 47 | 0.1451 |
| 48 | 48.9546 |
| 49 | 14.6239 |
| 50 | 1.8668 |
| 51 | 6.3675999999999995 |
| 52 | 5.8827 |
| 53 | 2.5181999999999998 |
| 54 | 3.2382 |
| 55 | 6.843299999999999 |
| 56 | 1.4485 |
| 57 | 0.7797000000000001 |
| 58 | 0.7870999999999999 |
| 59 | 0.4594 |
| 60 | 0.2322 |
| 61 | 0.1137 |
| 62 | 0.031599999999999996 |
| 63 | 0.0759 |
| 64 | 0.0362 |
| 65 | 0.008 |
| 66 | 0.0087 |
| 67 | 0.034699999999999995 |
| 68 | 0.0478 |C48 in RTp
50
50
30
A70 in HR2
40
40
A64 in HR2
Cleavage probability (%)
20
30
30
R67 in HR2
20
20
A51 in RTp & A64 in HR2
10
A64 in HR2
10
10
0
0
0
1
10
20
30
40
50
60
70
80
1
10
20
30
40
50
60
70
1
10
20
30
40
50
60
(aa)
(D)
(E)
Green
(sGFP)
Red
(mCherry)
Merged
(green + red)
Blue
(chlorophyll)
DIC
(White)
St
PG
RTp-HR2f-sG
/OsPSY2:mC
PG
St
PG
RTp-HR2b-sG
/OsPSY2:mC
RTp-HR2b-sG
RTp-HR2f-sG
RTp-HR2-sG
RTp-sG
(kDa)
70
Actin
43
28
Anti-GFP
Anti-mCherry
